# Immune-mediated insights into clinical and specific autoantibodies in acute and chronic immune-mediated nodo-paranodopathies

**DOI:** 10.1055/s-0045-1805073

**Published:** 2025-03-19

**Authors:** Marcus Vinícius Magno Gonçalves, Pedro José Tomaselli, Wilson Marques Junior

**Affiliations:** 1 Universidade da Região de Joinville, Curso de Medicina, Departamento de Neurologia, Joinville SC, Brazil.; 2 Universidade de São Paulo, Faculdade de Medicina de Ribeirão Preto, Departamento de Neurociências e Ciências do Comportamento, Ribeirão Preto SP, Brazil.; 3 Universidade de São Paulo, Faculdade de Medicina de Ribeirão Preto, Instituto Nacional de Ciência e Tecnologia Translacional em Medicina (INCT-TM), Ribeirão Preto SP, Brazil.

**Keywords:** Neuropathies, Neuritis

## Abstract

The recognition of the molecular structures, namely the node of Ranvier and the axonal regions surrounding it (the paranode and juxtaparanode), as the primary target for specific autoantibodies has introduced a new site for neurological location (microtopographic structures), in contrast to the prevailing understanding, in which lesions to neural macrostructures (roots, nerves, and/or plexus) were the focus of semiologists and electrophysiologists for topographic, syndromic, and nosological diagnoses. Therefore, there was a need to understand and characterize the components of these neural microstructures that are grouped in small regions within the nerve to optimize clinical and therapeutic reasoning.

## INTRODUCTION

Immune-mediated neuropathies represent a highly-heterogeneous group of neurological disorders in which an abnormal immune response targeting self-antigens within the peripheral nervous system (PNS) results in damage and, ultimately, nerve dysfunction. The 8-week progression period marks the boundary between the acute and chronic classification of these disorders. 1

From a practical standpoint, the acute presentations encompass Guillain-Barré syndrome (GBS) and its variants, while the chronic presentations include multifocal motor neuropathy (MMN), and chronic inflammatory demyelinating polyradiculoneuropathy (CIDP) and its variants. However, more recently, nodo-paranodopathies have also been included among immune-mediated neuropathies, with both acute and chronic forms exhibiting a behavior distinct from those of the classic etiologies previously mentioned. 2 Although their clinical phenotype ultimately resembles that of GBS and CIDP, specific characteristics are consistently observed, including non-responsiveness to first-line immunotherapies that are routinely effective in GBS and CIDP.

**Figure 1:**
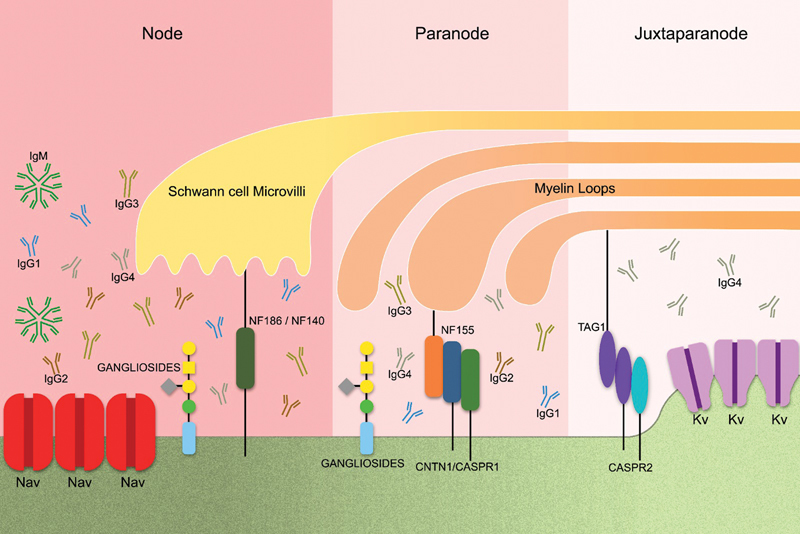
Nodal, paranodal, and juxtaparanodal regions with their main antigens, the presence of sodium channels in the nodal region, and of potassium channels in the juxtaparanodal regions. Predominance of immunoglobulin G1 (IgG1) to IG3 and IgM antibodies in the nodal region, and of IgG4 and 3 antibodies in the paranodal and juxtaparanodal regions.

The recognition of the molecular structures ( Figure 1 ), namely the node of Ranvier and the axonal regions surrounding it (the paranode and juxtaparanode), as the primary target for specific autoantibodies has introduced a new site for neurological location (microtopographic structures), in contrast to the prevailing understanding, in which lesions to macrostructures (roots, nerves, and/or plexus) were the focus of semiologists and electrophysiologists. Therefore, there was a need to understand and characterize the components of these neural microstructures that are grouped in small regions within the nerve. 3 4 5

Studies with animal models have facilitated the reproduction and have enhanced the characterization of the electrophysiological patterns observed in immune-mediated nodo-paranodopathies, while also enabling their correlation with the pathological findings observed in nerves through electron microscopy.

It is probable that the very first intriguing and, at some point, unexpected finding was that alterations initially classified as demyelinating based on electrophysiological patterns were instead caused by restricted uncoupling of nodo-paranodal regions and the absence of macrophages. 5 6 7 To unveil their mysteries, we will delve into them with meticulous scrutiny.

## MICROANATOMY OF NODE, PARANODE, AND JUXTAPARANODE REGIONS

The nodes of Ranvier are positioned along myelinated axons, in which interruptions in the myelin sheath expose the axolemma, which is covered only by the microvilli of Schwann cells. This region presents the highest density of voltage-gated sodium channels (VGSCs), mainly Nav1.6 and Nav1.1, providing a site for the generation and rapid propagation of action potentials. 8 Adjacent to the node of Ranvier lies the paranode, in which the myelin loops adheres to the axon, and the juxtaparanode, covered by compact myelin, in which most of voltage-gated potassium channel (VGKCs) are concentrated ( Figure 1 ).

The nodal region is composed of the extracellular matrix, proteins such as neurofascin 186 (NF-186), neurofascin 140 (NF-140), gliomedin and GM1, and GD1b and GQ1b gangliosides. 9

The paranode segment works as an electrical and biochemical barrier that restricts the mobility of ions and membrane proteins between the node and the juxtaparanode region. The paranode junction is composed of the proteins contactin 1 (CTCN1), contactin-associated protein 1 (CASPR-1) and neurofascin 155 (NF-155). The NF155 protein anchors at paranode loops of glia cells, while CTCN1 and CASPR-1 anchor at the axolemma. This glial-neural junction formed by this protein complex restricts mobility and stabilizes the nodal region. 6

Finally, in the juxtaparanode region, the areas adjacent to the paranodes beneath the myelin sheath have a high density of VGKCs associated with the presence of complex contactin-associated protein 2 (CASPR-2), which stabilize the potassium channels, 10 as well as of transiently-expressed glycoprotein-1, which is responsible for stabilizing the glial-neural framework in this location, in addition to the presence of GM1, GD1b and GQ1b gangliosides. 9

## PATHOPHYSIOLOGY

Autoantibodies targeting gangliosides (sphingolipids containing one or more sialic acid units), 11 or glycolipid proteins (primarily NF), play a crucial role in the pathophysiology of autoimmune neuropathies affecting nodes and paranodes. 12 This understanding reconciles clinical and electrophysiological concepts, as observed in the classic classification of neuropathies, with damage to macrostructures. 11 12

It is imperative to underscore that the designation *CIDP* for chronic neuropathies linked to immunoglobulin G4 (IgG4) antibodies targeting paranode epitopes (NF-155, CASPR-1, and CTCN1) is not conceptually correct and should not be used, mainly because chronic autoimmune nodo-paranodopathies are not primarily demyelinating neuropathies; rather, the target site of the underlying pathophysiological process includes specific portions of the nerve, enabling microstructural recognition. 5 The paranode axoglial junctions formed by the association of the proteins CNTCN-1, CASPR-1, and NF-155 play crucial roles in maintaining the paranode cytoarchitecture and in facilitating the neurophysiological propagation of nerve impulses along myelinated axons. 13 The autoantibodies against the antigens CNTCN-1, CASPR-1, and NF-155 belong to the IgG4 subclass. Immunoglobulin G4 accounts for approximately 5% of immunoglobulins, and it differs from other subclasses in several structural and functional aspects, 14 including its inability to activate the complement cascade and consequently the membrane attack complex, because it cannot bind to the first component of the complement cascade: C1q. 12 15

### Neurofascin-155

The NF-155 protein belongs to the L1 family of transmembrane cell adhesion molecules, typically presenting a well-conserved evolutionary structure of the protein domain, with 6 immunoglobulin domains and 5 fibronectin type-III domains, along with a transmembrane domain and a cytoplasmic domain comprising 113 amino acids. 16 17 The NF-155 protein is expressed in the paranodes, and its main function is the stabilization of the paranode cytoarchitecture. 18 Other isoforms of neurofascins (NF-166, NF-180, and NF-186) are equally involved in the dynamic mechanisms of synaptic stabilization, neural growth, and clustering of sodium channels. ^16^ Serological studies have shown that between 4 and 10% of the patients who were initially classified as having a chronic demyelinating neuropathy present positive serum autoantibody against this axoglial antigen. 19 20

The patients present with a characteristic clinical course, with symptom onset at a younger age (on average around 30 years), cerebellar tremor, dysarthria, and nystagmus, extremely high levels of protein in the cerebrospinal fluid, hypertrophy of the cervical and lumbar spinal roots, plexus, and peripheral nerves, and the possibility of demyelination of the central nervous system (CNS). 14 21 22 Electrophysiological abnormalities in visual evoked potentials occur in 70% of the patients during the course of the disease. 18

Nerve biopsy from these patients does not demonstrate the typical features of macrophage-mediated demyelination and onion-bulb remyelination, as observed in patients with classic CIDP. ^12^ Instead, it shows nodal widening and detached myelin loops due to node-paranode uncoupling and axonal degeneration. 22

Another distinctive feature in NF-155-seropositive patients is the presence of CNS lesions detected on brain magnetic resonance imaging (MRI) scans that resemble demyelination, 19 23 24 including clinical forms that meet the criteria for multiple sclerosis in up to 67.5% of the patients, 18 25 despite these questionable findings. 26 Regarding prognosis, NF-155-seropositive patients with involvement of both the CNS and PNS 27 have shown greater clinical disability and poor prognosis compared to those with exclusive PNS involvement. 19 Patients initially diagnosed with CIDP who present cerebellar tremor, associated CNS lesion on MRI suggestive of demyelination, and refractoriness to first-line immunotherapies should be tested for NF-155, as positivity could assist in a better therapeutic management due to the severe clinical evolution of this disease.

### Contactin-1

Autoantibodies against the CTCN1 antigen have been reported in 2.25 to 8.7% of the patients with immune-mediated chronic neuropathies. 3 15

The phenotype associated with CTCN1 is characterized by late onset (around 60 years of age), predominant motor neuropathy, untimely sensory ataxia, and axonal degeneration, along with a poor response to intravenous Ig (IVIg) treatment. 26 Miura et al. 28 described the clinical and serological characteristics of 13 Japanese patients initially diagnosed with CIDP that tested positive for CTCN1 antibodies (13 out of 533: 2.4%). Among these patients, 3 (23%) had an acute presentation, 1 (8%) developed cerebellar ataxia during the disease, 2 (15%) exhibited cerebellar tremor, and all (100%) had sensory ataxia upon examination. Regarding therapeutic response, 6 out of 10 patients treated with IVIg had poor response. 29 Therefore, elderly patients who meet the clinical and electrophysiological criteria for chronic demyelinating neuropathy and exhibit marked sensory ataxia, cerebellar tremor, and refractoriness to immunotherapy should be tested for CTCN1.

### Neurofascin-140 and neurofascin-186

Antibodies against 2 different isoforms of NF (NF-140/NF-186), have been described in up to 2% of the patients initially diagnosed with acute-onset CIDP or GBS. 29 Most patients experience severe disease, characterized by pronounced sensory ataxia, and demonstrate only a partial response to IVIg. Additionally, many of these individuals have concomitant autoimmune conditions. 5 The therapeutic response in some NF1-40-/NF-186-positive patients suggests that complement pathway activation is not the sole mechanism of action of human Ig in these patients. 27

## CASPR-1

The neuropathy in patients who tested positive for CASPR-1 typically begins around the age of 30 years, similar to patients with NF-155. It is a rapidly-progressive disease with proximal and distal weakness, lancinating neuropathic pain, and no response to IVIg. 26 The CASPR-1 antigen is an axonal-cell adhesion protein associated with contactin in the paranode region, which, together with CNTCN-1, binds to NF-155. These three proteins form the anchoring complex in the paranode region. As CASPR-1 is essential for the homeostasis and stabilization of sodium channels in the nodal region and potassium channels in the juxtaparanodal region, its function is to facilitate high-speed nerve conduction and myelin homeostasis in the CNS and PNS. 30 Conversely, anti-CASPR-1 autoantibodies bound in the paranodal region at the dorsal root ganglia trigger severe pain symptoms in these patients. 31

Nerve biopsy from patients with positive CASPR-1 antibodies reveals severe axonal degeneration and no onion bulb, indicating a different characteristic from the demyelination commonly found in the classic forms of CIDP. The myelin sheath is primarily less affected compared to the node and paranode regions, which undergo a decoupling of cytoarchitecture. Chronic immune-mediated neuropathies presenting with refractory neuropathic pain and lack of response to first-line immunotherapies should be tested for CASPR-1, as positivity could assist in better therapeutic choices due to the pronounced degree of axonal loss and morbidity from neuropathic pain in the course of this disease. 31 32

The availability of plasma autoantibodies 33 for the assessment of patients with an initial diagnosis of CIDP refractory 34 to first-line treatments or for those presenting signs and symptoms of CNS involvement remains limited in the clinical practice in Brazil. Establishing a diagnosis continues to be one of the major challenges in the therapeutic decision-making for patients with an initial CIDP diagnosis, 35 36 as well as for those with atypical CNS demyelinating diseases, 37 potentially increasing the therapeutic costs and morbidity due to diagnostic delays ( Table 1 ). 9 Another issue is that current treatment algorithms do not adequately address the underlying pathogenic heterogeneity of chronic immune-mediated neuropathies.

**Table 1:** Subtypes of nodo-paranodopathies, including clinical phenotypes, antibody subtypes, and therapeutic response

Region/antigen	IgG class and subclass	Clinical course	Initial diagnosis	CNS involvement	Response to first line therapy	CSF protein	Other
Node NF-186/NF-140	IgG3, IgG4.	Acute/subacute onset, motor and sensory, with severe course.	GBS or acute CIDP subacute.	No.	Some patients respond to IVIg.	Possible cytological albumin dissociation.	Nephrotic syndrome may be associated.
Paranode NF-155	Mainly IgG4.	Acute/subacute onset, mostly in young adults. Predominant distal weakness, ataxia and tremor.	Early-onset CIDP.	Yes.	Poor response to IVIg, response to rituximab.	> 150 mg/dL.	Appendicular, cephalic, vocal or tongue tremor may be associated. Root, plexus and/or nerve hypertrophy on MRI or US and MS-like on CNS MRI.
Paranode CTCN1	Mainly IgG4.	Acute/subacute onset, rapidly-progressive and severe. Predominant distal weakness, ataxia, and tremor.	Late-onset CIDP with pronounced sensory ataxia.	Yes.	Poor response to IVIg, response to rituximab.	Normal or hypoproteinorrhaquia in association with nephrotic syndrome.	Nephrotic syndrome and cerebellar tremor may be associated.
Paranode CASPR1	IgG3, IgG4.	Acute onset, rapidly-progressive weakness with ataxia, tremor, cranial nerve involvement, and neuropathic pain.	Early-onset CIDP.	No.	Poor response to IVIg, response to rituximab.	Normal.	Refractory pain.
Node/Paranode GM1	IgM.	Chronic, prevalently upper-limb chronic, asymmetrical, motor neuropathy.	ALS or CIDP of the motor subtype.	No.	Responsive to IVIg.	Normal.	No.
Node/Paranode GD1b, GQ1b	IgM.	Chronic sensory ataxia often with a relapsing–remitting pattern. It may be accompanied by oculomotor and bulbar weakness. Weakness may coexist. (CANDA/CANOMAD)	CIDP.	No.	Responsive to IVIg and rituximab.	Normal.	No.
Pan-neurofascinopathies	IgG1/IgG3/IgG4.	Acute/subacute onset, severe monophasic course, autonomic dysfunction, nephrotic syndrome, and respiratory involvement.	“GBS explosive”.	No.	Poor response to IVIg, response to plasmapheresis and rituximab.	Possible cytological albumin dissociation.	Nephrotic syndrome and retroperitoneal fibrosis may be associated.

Abbreviations: ALS, amyotrophic lateral sclerosis; CANDA/CANOMAD, chronic sensory ataxic neuropathy with anti-disialosyl antibodies/chronic ataxic neuropathy ophthalmoplegia M-protein agglutination disialosyl antibodies syndrome; CASPR1, contactin-associated protein 1; CIDP, chronic inflammatory demyelinating polyradiculoneuropathy; CNS, central nervous system; CSF, cerebrospinal fluid; CTCN1, contactin 1; GBS, Guillain-Barré syndrome; Ig, immunoglobulin; NF, neurofascin.

Algorithmically, regarding chronic paranodopathies, 9 we suggest, according to Table 1 , that: young patients initially diagnosed with CIDP, but with a subacute onset without proximal weakness and early axonal loss, should be tested for NF-155; if they present excruciating pain, they should be tested for CASPR 1; and elderly patients with severe sensory ataxia without proximal weakness and early axonal loss should be tested for CTCN1 ( Table 1 ). Regarding acute phenotypes, patients with GBS-like and CIDP-A phenotypes refractory to immunotherapy, early axonal loss, absence of dysautonomia, and predominantly-distal weakness should be tested for NF-186 and NF-140 according to the guideline of the European Academy of Neurology/Peripheral Nerve Society joint Task Force Force–second revision. 1
